# Western Diet: Implications for Brain Function and Behavior

**DOI:** 10.3389/fpsyg.2020.564413

**Published:** 2020-11-23

**Authors:** Isabel López-Taboada, Héctor González-Pardo, Nélida María Conejo

**Affiliations:** Laboratory of Neuroscience, Department of Psychology, Instituto de Neurociencias del Principado de Asturias (INEUROPA), University of Oviedo, Oviedo, Spain

**Keywords:** Western diet, addiction, stress, cognition, gut–brain axis

## Abstract

The Western diet (WD) pattern characterized by high daily intake of saturated fats and refined carbohydrates often leads to obesity and overweight, and it has been linked to cognitive impairment and emotional disorders in both animal models and humans. This dietary pattern alters the composition of gut microbiota, influencing brain function by different mechanisms involving the gut–brain axis. In addition, long-term exposure to highly palatable foods typical of WD could induce addictive-like eating behaviors and hypothalamic-pituitary-adrenal (HPA) axis dysregulation associated with chronic stress, anxiety, and depression. In turn, chronic stress modulates eating behavior, and it could have detrimental effects on different brain regions such as the hippocampus, hypothalamus, amygdala, and several cortical regions. Moreover, obesity and overweight induce neuroinflammation, causing neuronal dysfunction. In this review, we summarize the current scientific evidence about the mechanisms and factors relating WD consumption with altered brain function and behavior. Possible therapeutic interventions and limitations are also discussed, aiming to tackle and prevent this current pandemic.

## Introduction

Obesity and overweight are characterized by an excess of body fat accumulation, which harms our health (Gregg and Shaw, 2017; Hales et al., 2018; World Health Organization, 2020). Both conditions are an escalating pandemic in the 21st century. The World Health Organization reports that obesity and overweight are major risk factors for several chronic diseases, such as diabetes, cardiovascular diseases, and cancer (Kachur et al., 2017; Kuroda and Sakaue, 2017; Salaün et al., 2017; Guo et al., 2018). In addition, obesity and overweight are independent risk factors for stroke, mild cognitive impairment, and dementias, such as Alzheimer’s disease and vascular dementia (Xu et al., 2011; Guo et al., 2016; Pedditzi et al., 2016; Rodríguez-Castro et al., 2019). Obesity and overweight were once considered a problem only in high-income countries, but these conditions are now growing dramatically all over the world, particularly in urban settings in both developed and developing countries (World Health Organization, 2020). During the last decades, the worldwide prevalence of obesity and overweight has increased markedly in adults and even more in children (Di Cesare et al., 2019; Garrido-Migue et al., 2019). It is widely acknowledged that the main cause of obesity and overweight is an imbalance between consumed and expended calories, which, moreover, is aggravated by a sedentary lifestyle (Apovian, 2016). Moreover, there are more complex causes of these conditions involving genetic (Goodarzi, 2018; Loos, 2018) and endocrine factors (Camacho and Ruppel, 2017) together with environmental factors, such as pollution (de Bont et al., 2019), diet composition, and its effects on insulin or leptin secretion (Hall and Guo, 2017).

One of the main reasons related to obesity and overweight is the current obesogenic environment with wide availability of highly palatable foods that may drive addiction-like behaviors or “food addiction” (Morris et al., 2015; Leigh and Morris, 2018). However, the clinical validity of the concept of food addiction is highly controversial, and “hedonic overeating” has been proposed from a biopsychosocial perspective to better describe a behavioral pattern of overconsumption of particular calorie-rich foods together with a tendency to minimize physical activity (sedentary lifestyle) mediated by normal (not pathological) adaptive brain-mediated hedonic/reward processes (“wanting” or anticipatory motivation and “liking” or experience of pleasure) modulated by an obesogenic environment (Finlayson, 2017). This kind of calorie-dense food enriched in simple carbohydrates and saturated fats is known as the Western diet (WD) pattern (Varlamov, 2017). Specifically, the nutritional characteristics of the WD pattern are a high intake of saturated fats and omega-6 fatty acids, reduced omega-3 fat intake, excessive amounts of refined sugars, and an overuse of salt (Cordain et al., 2005). The habitual consumption of a WD is associated with many health problems, both physical and psychological (Myles, 2014). Currently, more than 80% of deaths in Western countries are due to non-communicable diseases, including those associated with aging and diseases caused or influenced by the consumption of a WD, such as type 2 diabetes, overweight, obesity, and cardiovascular diseases (Christ et al., 2018).

This brief review has two main objectives. First, we want to evidence how the consumption of a WD not only causes obesity, but also adversely impacts behavior, cognition, and emotion. In addition, we aim to discuss the current scientific evidence for the crucial role on brain function and behavior of the gut–brain axis and microbiota.

## Obesity, Overweight, and WD

Although WD causes numerous adverse health effects, first we focus on the key problem of addiction (Rogers, 2017). The over-availability of highly rewarding foods might lead to addiction-like behaviors as happens with tobacco, alcohol, and psychoactive drugs (Ziauddeen and Fletcher, 2013; Chen et al., 2017). A growing body of research reports many similarities between conventional addiction disorders and consumption of highly palatable food typical of WD in both animals (Jacques et al., 2019) and humans (Pursey et al., 2015; Teasdale et al., 2020). Some populations self-diagnose themselves as food addicts because of the environment full of hyperpalatable products (Hardman et al., 2015; Ruddock and Hardman, 2018; Ruddock et al., 2019). There are many studies about binge-like behavior associating substance use disorders with obesity and WD consumption. Binge-type intake of fat is related to the development of drug-seeking and -taking behaviors as already reported (Puhl et al., 2011; Ruddock et al., 2018). These authors suggest that fat bingeing could predispose some individuals to show major addiction-like behaviors, leading to drug abuse. For example, excessive intake of highly palatable fatty food could induce excessive seeking behavior, leading to cocaine consumption (Wellman et al., 2007). These maladaptive behaviors remain in time because long-term changes in brain are produced (Corbit et al., 2012; Furlong et al., 2014). Highly palatable food consumption can alter hedonic regulation involving opioid receptors and lead to compulsive consumption of obesogenic foods. That is exacerbated by aversive processes. Negative or stressful experiences could lead to binge-like behavior associated with altered brain dopamine levels (Davis et al., 2009; Groesz et al., 2012). Thus, when people binge on such WD foods, there is an increased release of dopamine in the mesocorticolimbic system, leading to compensatory changes in receptor expression (Reichelt and Rank, 2017; Thanarajah et al., 2019). Accordingly, changes in behavior following cessation of access to prolonged palatable food or WD consumption have been associated with low levels of dopamine and low expression of dopamine receptors in rats (Volkow et al., 2008; Johnson and Kenny, 2010; Morris et al., 2015).

## WD and Behavior

At this point, it is worth mentioning the “vicious cycle” model relating WD intake with cognition (Hargrave et al., 2016; Yeomans, 2017; Davidson et al., 2019). It is known that the hippocampus, in addition to memory and other cognitive processes, plays an important role in making decisions as in the case of starting and finishing eating (Parent et al., 2014). High-fat and -sugar diets involve the excessive consumption of these kind of food products and may result in weight gain. Moreover, it could lead to hippocampal function disturbances that alter the normal control of ingestion, increasing overconsumption even more (Kanoski et al., 2010). When this overconsumption persists over time because of addictive behavioral patterns related to food rich in saturated fats and simple sugars, the hippocampal dysfunction worsens, producing a decreased cognitive state and inhibiting normal intake control (Kanoski and Davidson, 2011). Moreover, these specific nutrients could lead to oxidative stress in brain metabolism, producing neurochemical modifications in the hippocampus, cortex, and hypothalamus (Lizarbe et al., 2019). When the vicious cycle is established, hippocampal dysfunction not only affects appetite and decision making, but also begins to affect cognition (Beilharz et al., 2015).

Overweight and obesity are usually associated with poorer memory performance because, as mentioned above, overconsumption of WD rich in saturated fats and simple sugars impairs memory. Human studies have examined how WD impairs hippocampal function and ingestive behavior (Stevenson et al., 2020). Several animal studies have been performed using memory tasks showing that WD consumption impairs hippocampal-dependent memory tasks (Francis and Stevenson, 2013; Ke et al., 2020). For example, a recent meta-analysis included 41 studies in which different tasks to assess hippocampal-dependent spatial learning and memory were carried out, and they showed that high-fat and -sugar diets may impair hippocampal-dependent forms of cognition (Abbott et al., 2019). In addition, human studies have demonstrated that WD is associated with decreased overall volume of the left hippocampus in a study performed in 60–64-year-old men (Jacka et al., 2015). The explanation for these harmful hippocampal effects associated with neurodegenerative diseases and increased neurogenesis in the adult brain is currently being investigated (Loprinzi and Frith, 2018; Hartanto et al., 2019). High-fat and -sugar diets are detrimental for anxiety-related behaviors, spatial learning, memory, and neurogenesis in both rodents and humans (Ferreira et al., 2018). However, there is a discussion about the cause–effect relationship of obesity because the connection between brain health and obesity is bidirectional. Obesity not only leads to impaired cognitive function, but it is also possible that deficits in executive functions may predispose to obesity by promoting uncontrolled food intake and a sedentary lifestyle (Miller and Spencer, 2014; Favieri et al., 2019).

In this regard, we need to take into consideration the involvement of epigenetic mechanisms in cognition and reward behaviors (Perrone-Capano and Di Porzio, 2000). In the same way, there should be a close correlation between these epigenetic modifications on reward circuitry and changes in our hedonic control of feeding (Contreras et al., 2019). Several studies show that there is a direct relationship between a high-fat diet during the early stages of fetal development (Champagne and Meaney, 2001; Giriko et al., 2013) and higher rates of depression-like symptoms in young adult male rodents. However, there is currently no evidence of epigenetic memory in the brains of human offspring born to obese mothers (Contu and Hawkes, 2017), and many more studies are necessary to be able to contribute to establishing the existence of epigenetic mechanisms involved in obesity and its regulation in the central nervous system (CNS) and peripheral tissues. On the other hand, the association between obesity and systemic neuroinflammation has been demonstrated in numerous studies (Pistell et al., 2010; Castanon et al., 2014; Erion et al., 2014; Lasselin et al., 2014). It is known that high-fat diets increase the proliferation of macrophages (phagocytes), initiating immunological processes by inducing phagocytosis in adipose tissue. This process may induce a proinflammatory cytokine cascade, among other mechanisms, that further activates the immune system and would impair food metabolism by causing neuroinflammation (Wang et al., 2012; de Sousa Rodrigues et al., 2017; Seong et al., 2019). Neuroinflammation is also associated with depression and impaired cognitive function, and it produces a cluster of disorders that are encompassed in metabolic syndrome, a combination of diabetes, hypertension, and high risk of coronary heart diseases and stroke (Baumgarner et al., 2014; Guillemot-Legris and Muccioli, 2017). Biological markers of both peripheral inflammation and neuroinflammation, such as C-reactive protein and cytokine levels, have been associated with depression and anxiety in several studies (Copeland et al., 2012; Kim et al., 2016; Köhler-Forsberg et al., 2017; Rossi et al., 2017). Other sources of neuroinflammation linked to obesity would be the high levels of endotoxins and inflammatory cytokines or reduced levels of anti-inflammatory commensal gut bacteria species associated with altered microbiota diversity (dysbiosis) in this population (Noble et al., 2017b). In addition, obesity is also related to central inflammation in vulnerable brain regions, such as the hypothalamus. The hypothalamus is an appetite regulator, and it is responsible for eating behavior. Hypothalamic inflammation deregulates brain energy homeostasis causing cognitive impairment because it can induce insulin resistance and leptin disbalance in the hypothalamus, which triggers overconsumption and weight gain (De Souza et al., 2005; Posey et al., 2009; Seong et al., 2019). Consumption of a fat-rich diet activates a pro-inflammatory response and increases proinflammatory cytokines (Puig et al., 2012).

## WD and Stress

The hypothalamic-pituitary-adrenal (HPA) axis mediates the stress response. There are some studies in which pro-inflammatory cytokines are elevated in the hypothalamus using rodent models of anxiety and depression, showing a direct relationship between neuroinflammation and the HPA axis (Wohleb et al., 2013). HPA axis dysregulation promotes impaired cognitive function because it induces the release of corticosterone that binds to hippocampal glucocorticoid (GR) and mineralocorticoid (MR) receptors, mediating inhibitory effects and causing repetitive stress (Makhathini et al., 2017). HPA axis dysregulation changes feeding behavior, leading to obesity because of hypercortisolism that increases the intra-adipose tissue GRs contributing to metabolic disease (Masuzaki et al., 2001). Thus, hypothalamic circuits and their functional connectivity with other brain regions (such as the hippocampus or the amygdala) are altered with neuroinflammation and obesity, producing disruptions to cognitive function and impairing hypothalamic satiety signals (Blundell et al., 2012; Hall et al., 2012; Goltz et al., 2018).

Stress can increase or decrease food intake in humans and animals. Stress triggers changes in the HPA axis, stimulating the production of hormones and peptides, such as leptin, ghrelin, insulin, or neuropeptide Y (Maniam and Morris, 2012; Huang et al., 2017; Toniazzo et al., 2018). These hormones establish neuropeptide circuits that regulate feeding control, and they activate brain regions involved in stress and motivation circuits during the stress response. These circuits that regulate energy intake have a control center in the hypothalamus, where the corticotrophin releasing hormone (CRH) neurons are located, controlling the HPA axis function and GR release. Furthermore, insulin and leptin regulate the appetite-satiety cycle, playing a neurotrophic factor role that affects memory and several behaviors by acting on the hypothalamus (Zanchi et al., 2017). Changes in stress-related hormones and neurotransmitters such as CRH, GRs, and norepinephrine could also sensitize brain reward pathways, including the nucleus accumbens and dorsal striatum, which would increase the drive to eat highly palatable foods typical of WD (Sharma et al., 2013). The severity of the stressor could also modulate food intake and body weight, associated with changes in the concentration of GR receptors (Sheriff et al., 2011; Quarta et al., 2017). Indeed, responses to stress can vary depending on the degree of HPA axis activation and GR release, which stimulates the release of orexigenic neuropeptides, increasing hunger, or anorexigenic neuropeptides, reducing it. It causes changes in leptin and insulin plasma levels (Maniam and Morris, 2012). This is even more relevant when the stress events happen during the early life period when organisms are most vulnerable. For instance, stress exposure during gestation or during postnatal periods modifies the offspring’s hypothalamic feeding circuits as previously explained and changes the expression of neuropeptides (Miller and Lumeng, 2018). Therefore, energy metabolism is altered in the offspring, predisposing them to obesity and metabolic-like syndromes in adulthood (Plagemann et al., 2009; Desai et al., 2014; Butruille et al., 2019). Epigenetic changes are produced in these new generations, such as modifications in gene promoters involved in stress and appetite regulation (Schneeberger et al., 2012; Schroeder et al., 2017). Moreover, early adverse childhood experiences could cause DNA methylations in genes associated with obesity (Kaufman et al., 2018; Rushing et al., 2020).

## The Gut–Brain Axis

The gut is considered as a “second brain” for the role it plays in influencing behavior and other basic CNS functions. The gut–brain axis refers to a bidirectional communication network, including the CNS, both brain and spinal cord, the autonomic nervous system (ANS), the enteric nervous system (ENS), and the HPA axis. The ENS establishes bidirectional communication between brain and gut. It is mediated by the vagus nerve (ANS) and the neuroimmune and neuroendocrine systems reaching the CNS (Chalazonitis and Rao, 2018). In addition, it is influenced by living gut microorganisms (microbiota), bacterial metabolites, cytokines, and neurotransmitters released in the bloodstream and directly affecting the ANS by the vagus nerve (Torres-Fuentes et al., 2017). Interestingly, it is recently reported that the gut–brain axis also contributes to sugar preference through vagal neurons specifically sensing glucose (Tan et al., 2020).

The term “microbiota” refers to the community of microorganisms that live in a particular place or organism that can be mostly symbiotic but also commensal or pathogenic. Chiefly, diet and environment determine the diversity of the human microbiota in the gut (Sekirov et al., 2010). Mice and human studies show the gut microbiota effect on host metabolism (Karl et al., 2017; Mithieux, 2018; Wang et al., 2018). It can increase or decrease energy yield from food and diet host-derived components and change metabolic pathways (Bäckhed et al., 2004; Ridaura et al., 2013). Probiotics and prebiotics play an important role in regulating gut microbiota composition (Wilson et al., 2020). Probiotics are live microorganisms that keep a balanced and diverse microbiota, and prebiotics are non-digestible fermented food ingredients that stimulate growth and benefit the gut microbiota (Markowiak and Ślizewska, 2017). Obese people usually show altered gut microbiota composition (dysbiosis), and their satiety-promoting hormones are also dysregulated (Zhang et al., 2015; Al-Mana and Robertson, 2018). A healthy microbiota represents balanced symbiosis, which means mutual benefits between bacteria and guest with inflammation levels decreased. However, an “unhealthy” microbiota produces dysbiosis, that is, a loss of composition and unbalanced microbiota with more pathobionts and less symbionts (Weiss and Hennet, 2017). External factors, such as diet, antibiotics, probiotics and prebiotics, stress, age, drug or alcohol intake, circadian rhythms, etc., modify the gut microbiota (Boulangé et al., 2016). Thus, it becomes a vicious cycle again (Tremaroli and Bäckhed, 2012). However, a change in diet clearly alters the gut microbiota, contributing to the host’s metabolic phenotype. Metabolites, such as short-chain fatty acids (SCFA) released by microbes, affect intestinal function and could alter brain function too (Maruvada et al., 2017).

There are several preclinical experiments that show the direct association between the gut microbiota and many maladaptive or pathological conditions, such as chronic stress, anxiety-like behavior, depression-like behavior, metabolic diseases, abnormal feeding behavior, etc., that is to say, these preclinical studies have identified the influence of gut microbiota on the CNS (Sandhu et al., 2017; Martin et al., 2018; Winter et al., 2018). The main communication pathways are mediated by molecules derived from bacteria, including SCFAs, secondary bile acids, and tryptophan metabolites. The latter metabolites influence behavior, such as 5-HTP (5-hydroxytryptophan) or serotonin (5-hydroxytryptamine) that modulate neurotransmitter release in the CNS affecting mood, emotions, appetite, anxiety, stress, etc. (O’Mahony et al., 2015). Bacteria interact with enteroendocrine cells (EEC), enterochromaffin cells, and the mucosal immune system through chemical signals (SCFAs, tryptophan metabolites, etc.). Then, these chemical signals may cross the intestinal barrier, being able to reach systemic circulation, and finally, they are able to cross the blood–brain barrier (BBB) (Haghikia et al., 2015). In addition, gut peptide hormones released by EEC are modulated by these microbiota-derived chemical signals and interact with several receptors of immune system cells and on vagus terminals in the gut (Okano-Matsumoto et al., 2011; Desbonnet et al., 2015). Gut peptide hormones released in systemic circulation, in turn, could modulate appetite, mood, or anxiety (Lach et al., 2018). The BBB is a selective, semipermeable border of endothelial cells whose function is to prevent the entry of substances to the CNS from the circulating blood (Obermeier et al., 2016). Gut microorganisms, stress, and inflammation can modulate the permeability of the intestinal barrier and the BBB. Therefore, gut–brain bidirectional communication is variable depending on the state of the host and their lifestyle habits (Grenham et al., 2011; Claesson et al., 2012; Valle Gottlieb et al., 2018; La-Ongkham et al., 2020).

Macronutrients and micronutrients of the diet directly contribute to the synthesis of metabolites by gut microbiota. Carbohydrates, proteins, and lipids are metabolized by the gut microbiota, and each one releases different substances. Some of these substances are SCFAs and bile acids that stimulate gut hormone secretion, modulating the CNS and food intake control (Dockray, 2014; Miquel-Kergoat et al., 2015). Furthermore, there are neurotransmitters and neuroactive substances produced by the gut microbiota that enter the circulatory system and cross the BBB, modulating cognition and emotion, neuroprotection, and neuropsychiatric conditions (Sandhu et al., 2017). As suggested previously, the gut microbiota relationship changes with age (Claesson et al., 2011; Rampelli et al., 2013; Biagi et al., 2016). Maternal and neonatal diet is critical for shaping the gut microbiota in the offspring. During lactation, infants fed on breast milk have a microbiota dominated by beneficial bacteria. However, formula-fed infants have more pathological bacteria and facultative anaerobic bacteria (Cerdó et al., 2018). When this formula diet is enriched in prebiotics, the bacteria composition improves in infants, increasing the number of beneficial bacteria, such as *Bifidobacteria* and *Lactobacillus* sp. and reducing bacterial species associated with pathogenesis (Ben et al., 2008; Borewicz et al., 2019). Later, when the offspring starts eating a solid diet during early life stages, a healthy diet (non-WD) keeps growth and development of the beneficial bacteria, and a WD pattern influences gut microbiota in early life (Noble et al., 2017a; Sandhu et al., 2017). Nevertheless, there are studies suggesting detrimental physiological effects caused by an unhealthy diet and altered microbiota can be rescued (Blanton et al., 2016). On the other hand, from adulthood to the elderly, it changes too. Elderly people usually experience reductions in microbiota diversity and composition because factors such as nutritional behavior, digestion, dentition, stress, and lifestyle are altered (Claesson et al., 2011).

A WD pattern rich in fats, sugar, and salt alters gut microbiota composition and is associated with obesity, chronic inflammation, allergies, diabetes, autoimmune disorders, depression, metabolic syndrome, and neuropsychiatric disorders (Martinez et al., 2017; Hintze et al., 2018; Zinöcker and Lindseth, 2018). WD is associated with low levels of beneficial bacteria and SCFAs. The most abundant SCFAs are butyrate, acetate, and propionate. In healthy conditions, SCFAs reinforce the BBB integrity, modulate neurotransmission, change the neurotrophic factor levels, and promote memory consolidation. However, when SCFAs are unbalanced because of diet, these functions are negatively altered (de Clercq et al., 2016; Silva et al., 2020). Unbalanced SCFA concentrations in gut lumen can increase intestinal permeability and induce systemic inflammation and insulin resistance due to the synthesis of pro-inflammatory molecules (Poroyko et al., 2016; Feng et al., 2018). Chronic inflammation in obese people promotes clinical progression to metabolic syndrome and some pathologies, such as type 2 diabetes or hepatic steatosis (Ellulu et al., 2015; Kim et al., 2019; Polyzos et al., 2019; Yu et al., 2019). In addition, interactions of the gut–brain axis modulated by microbiota could increase the risk of anxiety, depression, and additional mental disorders (Kelly et al., 2016; Lach et al., 2018). Finally, the vicious cycle hypothesis of obesity is proposed because diet provides the substrate for the gut microbiota, and the microbiota importantly contributes to appetite and food intake through satiety neuropeptides, and last, the CNS mediates the preference for food intake again, and the cycle starts once more (Hargrave et al., 2016; Sandhu et al., 2017; Davidson et al., 2019).

## Future Directions

Obesity and overweight could be considered in an evolutionary context with different theories about their genetic causes. Genome-wide association studies (GWAS) have identified more than 300 single-nucleotide polymorphisms associated with adiposity traits (Gunstad et al., 2006; Larsen et al., 2012; Goodarzi, 2018). In particular, genes near loci regulating the body mass index (BMI) show increased expression in the CNS, suggesting that BMI is largely regulated by hypothalamic regions involved in energy intake (Willer et al., 2009; Speliotes et al., 2010; Blundell et al., 2012; Locke et al., 2015). Novel pharmacological approaches could be developed to prevent or treat obesity based on the molecular targets reported by gene polymorphisms found in GWAS. However, additional GWAS studies are required by considering more complex energy balance-related traits and taking into account the individual variations (Müller et al., 2018; Speakman et al., 2018). Moreover, many studies carried out in both rodents and humans have related a worse cognitive state and obesity trends in subjects whose mothers followed a WD during pregnancy and lactation. In this regard, bad nutritional habits could be transmitted through epigenetic mechanisms from mothers to offspring (Kang et al., 2014). Interestingly, neuroinflammation and systemic inflammation triggered by altered microbiota composition reported in obese and overweight people could be the target of novel pharmacological treatments and lifestyle interventions (Guillemot-Legris and Muccioli, 2017; Solas et al., 2017; Janakiraman and Krishnamoorthy, 2018).

In addition, sex and gender differences on programming of behavior, brain development, and metabolism by diet during early life should be addressed in future studies. In this regard, exposure to a high-fat and -sugar diet prenatally and during early life, together with early life psychosocial stress, induce sex-dependent effects on adult metabolism, neuroinflammation, altered brain energy metabolism, and monoaminergic activity as recently reported by us and other research groups (Dearden et al., 2018; González-Pardo et al., 2020).

On the other hand, in the last few years, the brain–gut axis has become an important hidden physiological pathway. Scientific evidence indicates that the gut microbiota may be a target for treating metabolic diseases, using prebiotics, probiotics, and healthy diets. But there is another alternative, the fecal microbiota transplant (FMT). It consists of altering gut microbiota through transplantation from stool of healthy individuals. The healthy donor microbes are isolated from fecal sediment and are administered to a receptor whose microbiota is altered because of any pathologies, such as neurodegenerative diseases, depression, irritable bowel syndrome, autism, etc. (Tremaroli and Bäckhed, 2012; Vindigni and Surawicz, 2017; Wang et al., 2019). However, the possible long-term effects of FMT have not been analyzed systematically, and the methods of FMT have not yet been standardized. FMT after chronic unpredictable mild stress in mice caused, in untreated recipient mice, anxiety- and depression-like behaviors and increased neuroinflammation like the donor mice (Li et al., 2019). In addition, most studies with human microbiota-associated rodents report transfer of pathological phenotypes to recipient animals, including obesity and/or altered behavior, but extrapolation to humans or inferring causality in these studies is still uncertain (Walter et al., 2020). In this regard, clinical trials with FMT for major depression and/or anxiety have reported no effects or return to baseline depression scores after 3, 5, or 6 months (Mizuno et al., 2017; Mazzawi et al., 2018; Huang et al., 2019). The main limitation of clinical trials of FMT for mental disorders is the small sample size, the long-term efficacy, and the still undefined concept of “healthy microbiota” with wide variations in the taxonomic composition of gut microbiota among “healthy” individuals (Chinna Meyyappan et al., 2020). In this regard, FMT for obesity and its metabolic effects has not been successful in recent randomized clinical trials despite host microbiota engraftment (Yu et al., 2020) nor reduced BMI in obese patients (Allegretti et al., 2020). There is an ongoing randomized clinical trial of FMT for obesity in adolescents that will assess long-term BMI changes, adiposity, and insulin sensitivity in male and female participants (Leong et al., 2019). We need more studies in the near future to consider FMT as a target for therapeutic intervention of many diseases.

One of the main interventions for overweight and obesity, besides diet modification, should be to promote a healthy lifestyle. It should be considered that obesity is not solely a metabolic disorder, but a multifactorial disease (Hruby et al., 2016; Chooi et al., 2019; Rohde et al., 2019; Hu et al., 2020). The best way to improve the situation is to provide education in healthy diets and to raise awareness about WD diets and sedentary lifestyle because obesity is a 21st-century pandemic being associated with cognitive and mental health problems, non-communicable diseases, and premature death (Lach et al., 2018; Cena and Calder, 2020).

## Author Contributions

IL-T wrote the draft manuscript. HG-P and NC reviewed and edited the manuscript. All authors contributed equally to the idea of the manuscript and approved the final manuscript.

## Conflict of Interest

The authors declare that the research was conducted in the absence of any commercial or financial relationships that could be construed as a potential conflict of interest.
